# Supplementary material for: Mechanical coordination between anaphase A and B drives asymmetric chromosome segregation
Source: bioRxiv. 2025 Oct 4:2025.10.02.680008. Preprint. [Version 1] doi: 10.1101/2025.10.02.680008 (PMC12621997; doi:10.1101/2025.10.02.680008)
Supplement: Supplement 1 — Figure S1. Workflow for generating composite kymographs and color-coded graphs. (A) Top left: kymograph, aligned on the anterior spindle pole, at 10-second intervals from a zygote expressing mCherry-HIS-58H2B (magenta), GFP::TBG-1γ-tubulin (green) and GFP::AIR-2AuroraB (green), recorded from 0 to 100 seconds post-anaphase onset. Top right: Corresponding kymograph aligned on the posterior spindle pole. Middle: Cropped kymographs along the anterior (left) and posterior (right) set of segregating chromosomes. Bottom: Composite kymograph generated by joining the cropped kymographs to allow visualizing chromosome displacement. Scale bar, 5 μm. (B) Workflow for generating the color-coded graphs. For each timepoint (from 0 to 100 seconds after anaphase onset), the chromosome displacement was color-coded from dark blue (0.5 μm anti-poleward displacement) to red (2 μm poleward displacement) and mounted vertically as a kymograph, with the anterior pole on the left and the posterior pole on the right. The width of the uncropped color-coded graph indicates the spindle length at anaphase onset. For easy comparison, all color-coded graphs were arbitrarily cropped to the same width. Chromosomes are depicted by vertical black lines. Chromosome segregation driven by chromosome displacement is visualized as the white space between black lines. (C-D) Composite kymographs (C) and their corresponding color-coded graphs (D) for zygotes expressing TBG-1γ-tubulin::mScarlet and mCherry::HIS-11H2B (left), TBG-1γ-tubulin::mCherry and mCherry::HIS-58H2B (middle) and TBG-1γ-tubulin::GFP and GFP::HIS-11H2B (right). The sample size is indicated at the bottom of each color-coded graph. Scale bar, 2 μm. All error bars represent the 95% confidence interval. Figure S2. Targeted mini-screen to identify proteins involved in regulating chromosome displacement. (A) Quantification of the mean chromosome displacement over time from anaphase onset for the anterior (circles) and posterior (squares) chromosom [file media-1.pdf]

Figure S1

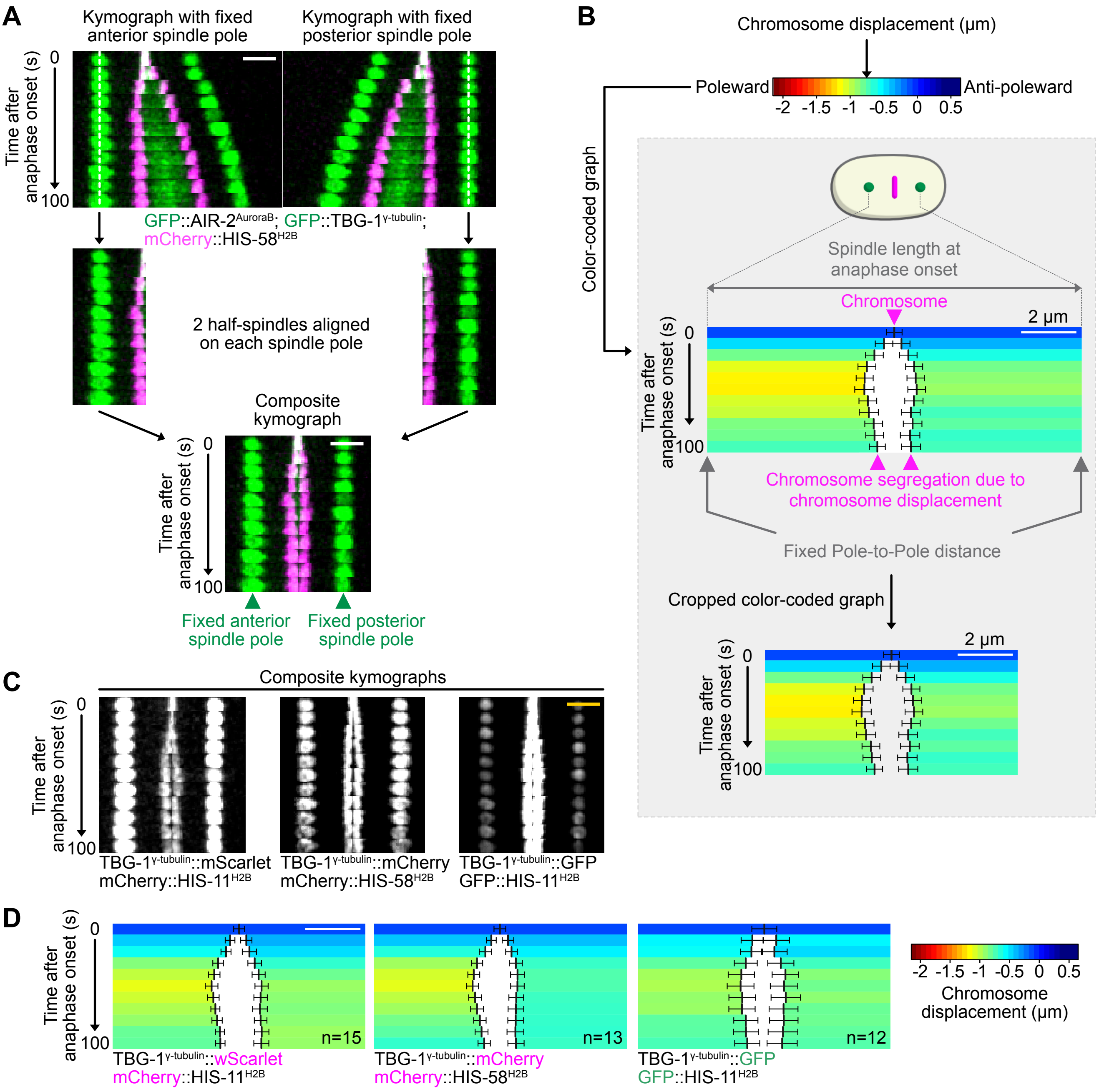
A
Kymograph with fixed anterior spindle pole
Kymograph with fixed posterior spindle pole
Time after anaphase onset (s)
0
100
GFP::AIR-2AuroraB; GFP::TBG-1γ-tubulin;
mCherry::HIS-58H2B
2 half-spindles aligned on each spindle pole
Composite kymograph
Fixed anterior spindle pole
Fixed posterior spindle pole
B
Chromosome displacement (μm)
Poleward
Anti-poleward
-2 -1.5 -1 -0.5 0 0.5
Color-coded graph
Spindle length at anaphase onset
Chromosome
2 μm
Chromosome segregation due to chromosome displacement
Fixed Pole-to-Pole distance
Cropped color-coded graph
C
Composite kymographs
TBG-1γ-tubulin::mScarlet
mCherry::HIS-11H2B
TBG-1γ-tubulin::mCherry
mCherry::HIS-58H2B
TBG-1γ-tubulin::GFP
GFP::HIS-11H2B
D
n=15
n=13
n=12
TBG-1γ-tubulin::wScarlet
mCherry::HIS-11H2B
TBG-1γ-tubulin::mCherry
mCherry::HIS-58H2B
TBG-1γ-tubulin::GFP
GFP::HIS-11H2B
Chromosome displacement (μm)

Figure S2

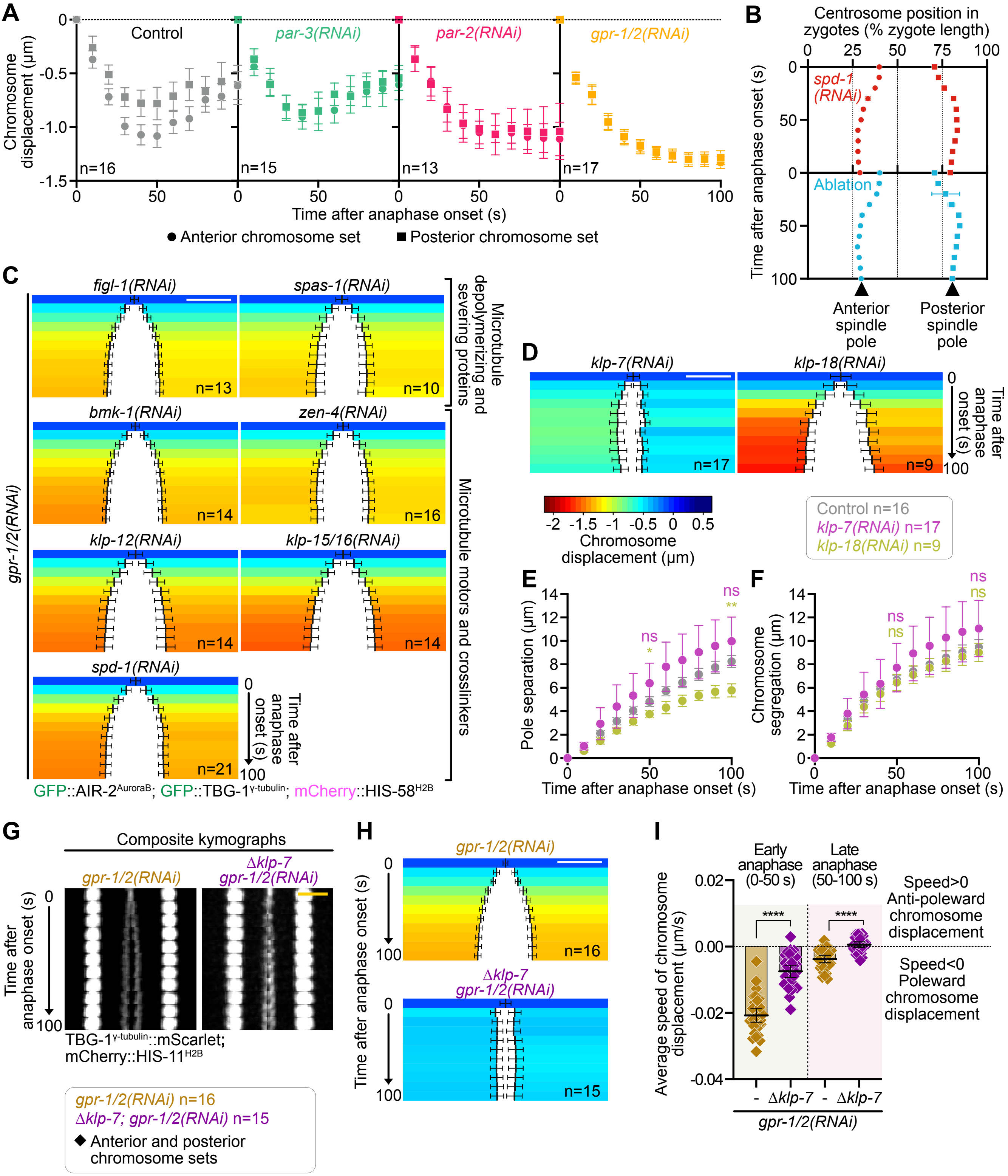

A
Control
par-3(RNAi)
par-2(RNAi)
gpr-1/2(RNAi)
Chromosome displacement (µm)
n=16
n=15
n=13
n=17
Time after anaphase onset (s)
● Anterior chromosome set
■ Posterior chromosome set
B
Centrosome position in zygotes (% zygote length)
spd-1 (RNAi)
Ablation
Time after anaphase onset (s)
Anterior spindle pole
Posterior spindle pole
C
figl-1(RNAi)
spas-1(RNAi)
n=13
n=10
Microtubule depolymerizing and severing proteins
bmk-1(RNAi)
zen-4(RNAi)
n=14
n=16
klp-12(RNAi)
klp-15/16(RNAi)
n=14
n=14
Microtubule motors and crosslinkers
spd-1(RNAi)
n=21
Time after anaphase onset (s)
gpr-1/2(RNAi)
GFP::AIR-2AuroraB; GFP::TBG-1γ-tubulin; mCherry::HIS-58H2B
D
klp-7(RNAi)
klp-18(RNAi)
n=17
n=9
Time after anaphase onset (s)
Chromosome displacement (µm)
Control n=16
klp-7(RNAi) n=17
klp-18(RNAi) n=9
E
Pole separation (µm)
ns
*
ns
**
Time after anaphase onset (s)
F
Chromosome segregation (µm)
ns
ns
ns
ns
Time after anaphase onset (s)
G
Composite kymographs
gpr-1/2(RNAi)
Δklp-7 gpr-1/2(RNAi)
Time after anaphase onset (s)
TBG-1γ-tubulin::mScarlet; mCherry::HIS-11H2B
gpr-1/2(RNAi) n=16
Δklp-7; gpr-1/2(RNAi) n=15
◆ Anterior and posterior chromosome sets
H
gpr-1/2(RNAi)
n=16
Δklp-7 gpr-1/2(RNAi)
n=15
Time after anaphase onset (s)
I
Early anaphase (0-50 s)
Late anaphase (50-100 s)
Average speed of chromosome displacement (µm/s)
****
****
Speed>0 Anti-poleward chromosome displacement
Speed<0 Poleward chromosome displacement
- Δklp-7 - Δklp-7
gpr-1/2(RNAi)

Figure S3

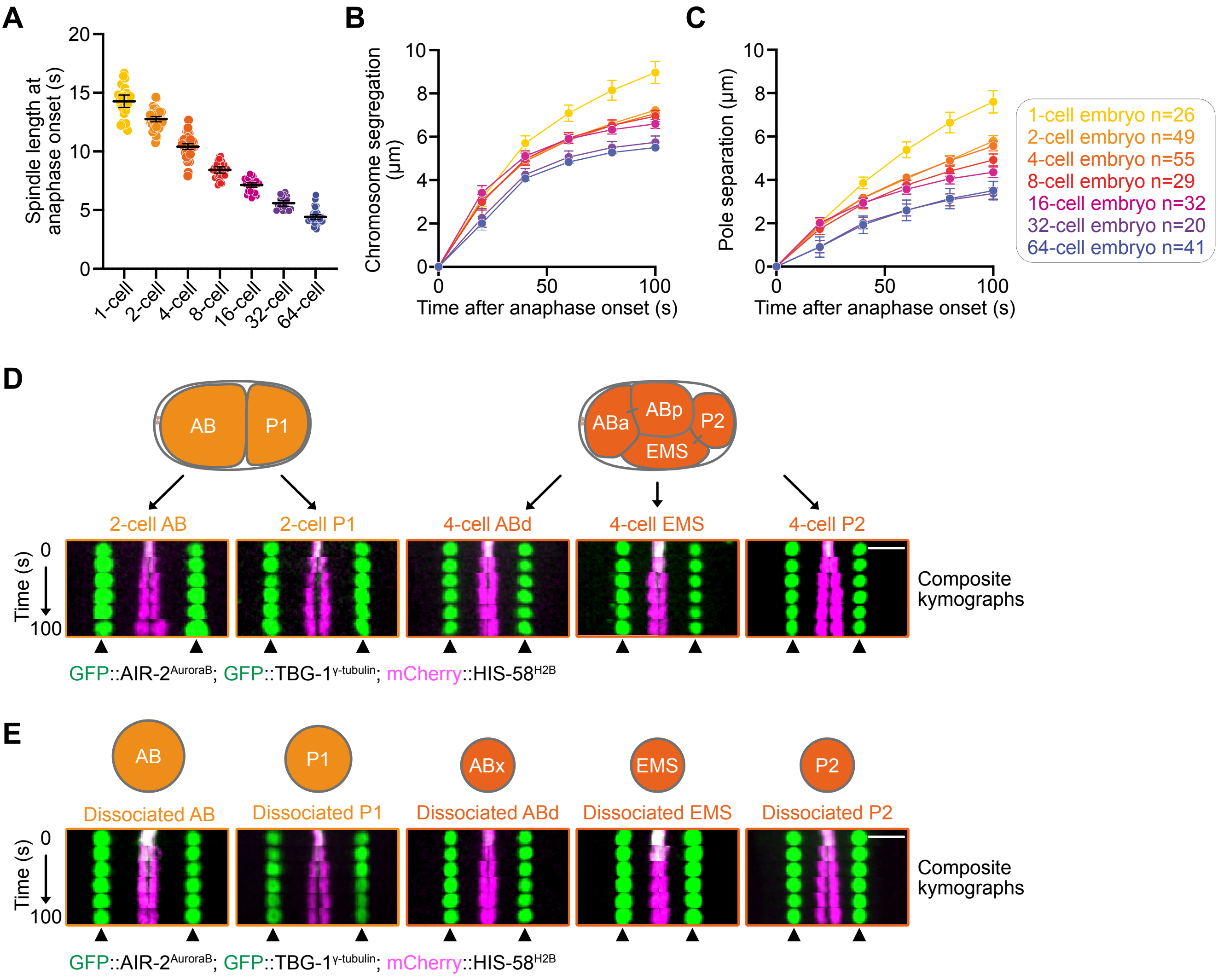
A
Spindle length at anaphase onset (s)
20
15
10
5
0
1-cell
2-cell
4-cell
8-cell
16-cell
32-cell
64-cell
B
Chromosome segregation (μm)
Time after anaphase onset (s)
C
Pole separation (μm)
Time after anaphase onset (s)
1-cell embryo n=26
2-cell embryo n=49
4-cell embryo n=55
8-cell embryo n=29
16-cell embryo n=32
32-cell embryo n=20
64-cell embryo n=41
D
AB
P1
ABa
ABp
P2
EMS
2-cell AB
2-cell P1
4-cell ABd
4-cell EMS
4-cell P2
Time (s)
0
100
Composite kymographs
GFP::AIR-2AuroraB; GFP::TBG-1γ-tubulin; mCherry::HIS-58H2B
E
AB
P1
ABx
EMS
P2
Dissociated AB
Dissociated P1
Dissociated ABd
Dissociated EMS
Dissociated P2
Time (s)
0
100
Composite kymographs
GFP::AIR-2AuroraB; GFP::TBG-1γ-tubulin; mCherry::HIS-58H2B

Supplementary Table 1: Strains used in this study

Strain	Genotype	Source
JCC56	unc-119(ed3)III?; ltIs37[pAA64; Ppie-1/mCherry::his-58; unc-119 (+)]IV, ltIs14[pASM05; Ppie-1/GFP-TEV-STag::air-2; unc-119 (+)]IV; ddIs6[Ppie-1/GFP:: tbg-1; unc-119(+)]V	Maton et al., 2015
JDU570	ijmSi125[pJD768; Pmex-5/tbg-1::mScarlet; mCherry::his-11; cb-unc-119(+)]II	This study
JDU585	ijmSi125[pJD768; Pmex-5/tbg-1::mScarlet; mCherry::his-11; cb-unc-119(+)]II; klp-7(ijm13)III	This study
JDU711	ltSi569[oxTi185; pOD1110/pSW008; CEOP3608 tbg-1::mCherry; cb-unc-119(+)]I; unc-119(ed3)III?; ltIs37[pAA64; pie-1/mCherry::his-58; unc-119 (+)]IV	This study
OD1702	unc-119(ed3)III; ltSi560 [pPLG014; Pmex-5/GFP::his-11::tbb-2_3'UTR, tbg-1::gfp::tbb-2_3'UTR; cb-unc-119(+)]V	Kim et al., 2015

Table S2: Oligonucleotides used for dsRNA synthesis

Gene	Oligonucleotide 1 (5’ → 3’)	Oligonucleotide 2 (5’ → 3’)	Template	[C] (mg/ml)
spd-1 (Y34D9A.4)	TAATACGACTCACTATAGGctcttcccagtaaaggcgttcg	AATTAACCCTCACTAAAGGtttagccacgggctcc atcttcg	gDNA	1.28
gpr-1/2 (F22B7.13 / C38C10.4)	TAATACGACTCACTATAGGagcatgtgattccacacgtc	AATTAACCCTCACTAAAGGtctggcagcagacag ttcag	gDNA	1.74
par-2 (F58B6.3)	TAATACGACTCACTATAGGccggctccagagtgtcc	AATTAACCCTCACTAAAGGgccgtcgcccactgt cg	gDNA	1.32
par-3 (F54E7.3)	TAATACGACTCACTATAGGagacttctagagatcaatgg	AATTAACCCTCACTAAAGGttgatgtgctgtggat cagc	gDNA	0.91
figl-1 (F32D1.1)	TAATACGACTCACTATAGGaatggcagtacaacaatctcc	AATTAACCCTCACTAAAGGctcaatgtgaccagt ggaatg	cDNA	1.15
spas-1 (C24B5.2)	TAATACGACTCACTATAGGttagcaaccgaaacttcgaga g	AATTAACCCTCACTAAAGGgcaagtcttccgatg catcag	cDNA	1.52
klp-7 (K11D9.1)	TAATACGACTCACTATAGGaaaaaggtgtggggcaagtt	AATTAACCCTCACTAAAGGgacacgggtgttcga gacta	cDNA	1.30
bmk-1 (F23D12.8)	TAATACGACTCACTATAGGagctcaactgatgaccctac	AATTAACCCTCACTAAAGGgccatttcgcgaattc gatc	gDNA	2.02
zen-4 (M03D4.1)	TAATACGACTCACTATAGGattggagctgttggatgagc	AATTAACCCTCACTAAAGGaattggttatggctcc gaga	cDNA	1.50
klp-12 (T01G1.1)	TAATACGACTCACTATAGGacactgaaactgaacgagatc g	AATTAACCCTCACTAAAGGttctacaatttccacc attac	gDNA	1.14
klp-15/16 (M01E11.6 / C41G7.2)	TAATACGACTCACTATAGGtgcttgtcgtcctccgtctcgtt tgc	AATTAACCCTCACTAAAGGtggattcagcgaaga gaaatgcagc	gDNA	1.31
klp-18 (C06G3.2)	TAATACGACTCACTATAGGgttgatgacctccgtgtcct	AATTAACCCTCACTAAAGGtgacgagaaacaga agtttgaca	gDNA	1.19
